# Interaction Among Sex, Aging, and Epigenetic Processes Concerning Visceral Fat, Insulin Resistance, and Dyslipidaemia

**DOI:** 10.3389/fendo.2019.00496

**Published:** 2019-07-17

**Authors:** Ana Arpón, Fermín I. Milagro, José L. Santos, Marta García-Granero, José-Ignacio Riezu-Boj, J. Alfredo Martínez

**Affiliations:** ^1^Department of Nutrition, Food Sciences and Physiology, University of Navarra, Pamplona, Spain; ^2^Centre for Nutrition Research, University of Navarra, Pamplona, Spain; ^3^Centro de Investigación Biomédica en Red Fisiopatología de la Obesidad y Nutrición (CIBERobn), Instituto de Salud Carlos III, Madrid, Spain; ^4^Navarra Institute for Health Research (IdiSNa), Pamplona, Spain; ^5^Department of Nutrition, Diabetes and Metabolism, School of Medicine, Pontificia Universidad Católica de Chile, Santiago, Chile; ^6^Department of Biochemistry and Genetics, University of Navarra, Pamplona, Spain; ^7^Precision Nutrition and Cardiometabolic Health Program, Madrid Institute for Advanced Studies (IMDEA), IMDEA Food, Madrid, Spain

**Keywords:** DNA methylation, visceral adipose tissue, C-reactive protein, HDL-cholesterol, TyG index

## Abstract

The distribution of adipose tissue is influenced by gender and by age, shifting from subcutaneous to visceral depots with longevity, increasing the development of several aging-related diseases and manifestations such as obesity, metabolic syndrome, and insulin resistance. Epigenetics might have an important role in aging processes. The aim of this research was to investigate the interactions between aging and epigenetic processes and the role of visceral adipose tissue, insulin resistance, and dyslipidaemia. Two different study samples of 366 and 269 adult participants were analyzed. Anthropometric, biochemical (including the triglycerides-glucose (TyG) index), and blood pressure measurements were assessed following standardized methods. Body composition measurements by Dual-energy X-ray absorptiometry (DXA) were also performed for the second sample. Methylation data were assessed by Infinium Human Methylation BeadChip (Illumina) in peripheral white blood cells. Epigenetic age acceleration was calculated using the methods DNAmAge (AgeAcc) and GrimAge (AgeAccGrim). Age acceleration (AgeAccGrim) showed better correlations than AgeAcc with most of the measured variables (waist circumference, glucose, HOMA-IR, HDL-cholesterol, triglycerides, and TyG index) for the first sample. In the second sample, all the previous correlations were confirmed, except for HOMA-IR. In addition, many of the anthropometrical measurements assessed by DXA and C-reactive protein (CRP) were also statistically associated with AgeAccGrim. Associations separated by sex showed statistically significant correlations between AgeAccGrim and HDL-cholesterol or CRP in women, whereas, in men, the association was with visceral adipose tissue mass DXA, triglycerides and TyG index. Linear regression models (model 1 included visceral adipose tissue mass DXA and TyG index and model 2 included HDL-cholesterol and CRP) showed a significant association for men concerning visceral adipose tissue mass DXA and TyG index, while HDL-cholesterol and CRP were associated in women. Moreover, structural equation modeling showed that the TyG index was mediating the majority of the visceral adipose tissue mass action on age acceleration. Collectively, these findings showed that there are different mechanisms affecting epigenetic age acceleration depending on sex. The identified relationships between epigenetic age acceleration and disease markers will contribute to the understanding of the development of age-related diseases.

## Introduction

Adipocytes are not only cells responsible for fuel storage triggered by a chronic imbalance between energy intake and energy expenditure, but they also participate in hormonal regulatory functions as well as in the synthesis of several endocrine/autocrine factors implicated in body homeostasis (1).

Excessive adipose tissue mass, particularly in the visceral compartment, has been related to hyperglycaemia, hypertension, hypercholesterolemia, and inflammatory processes (2). Indeed, obesity is associated with insulin resistance (IR), which, in turn, is also related to increased circulating levels of non-esterified fatty acids (3). This excess of lipids not only accumulates in different tissues, but it is also transported as very low-density lipoprotein triglycerides and secreted by the liver, leading to dyslipidaemia, which is a common feature of obesity and diabetes (4). These metabolic dysregulations are usually accompanied by changes in the endocrine balance (5) and in the secretory pattern of the adipose tissue (6, 7). Most of these metabolic processes are also related to aging, since nutrient-regulated pathways and energy balance may have a role in accelerating or delaying senescence (8).

On the other hand, the distribution of adipose tissue changes during the course of a lifetime, shifting from subcutaneous depots to intra-abdominal and ectopic fat deposition (9), and it is also influenced by sex (10). This fat/fuel redistribution has also been associated with the development of several aging-related diseases and manifestations, including obesity, metabolic syndrome and IR (11). Inadequate dietary habits and sedentarism, which are intimately related to increases in visceral fat, also contribute to the aging process, partly mediated by epigenetic mechanisms (12, 13).

Epigenetics involves a number of processes that modulate gene expression without altering the DNA sequence, such as DNA methylation, histone modifications and miRNAs (14). Several reports have related epigenetic signatures with longevity and aging. For example, Salas-Pérez et al., identified several differentially methylated sites that could be implicated in longevity and the development of metabolic disturbances (15). Furthermore, Horvath et al. have published several investigations relating DNA methylation with aging, and subsequently, developing different epigenetic clocks to measure “biological age” (16–18). Additionally, there is a general loss of histones in aging cells, and several modifications have been related to lifespan, such as histone methylation or histone acetylation (19). Moreover, miRNAs have also been involved in aging processes; for example, miR-34a has been described as an aging marker in several tissues and systems since it is upregulated in the aging heart, and its inhibition reduces cell death and fibrosis after an acute myocardial infarction (20).

The aim of this research was to investigate the interactions between aging and epigenetic processes, as well as the role of visceral adipose tissue, IR and dyslipidaemia on epigenetic age acceleration, together with putative mediating effects.

## Subjects and Methods

### Participants

This research was performed in two different study samples. The first study involved 366 adult participants from studies and cohorts belonging to the Methyl Epigenome Network Association (MENA) project such as DiOGenes-UNAV with *n* = 52 (21), OBEPALIP with *n* = 29 (22), Food4Me-UNAV with *n* = 39 (23), GEDYMET with *n* = 57 (24), ICTUS with n = 7 (25), NUGENOB-UNAV with *n* = 22 (26), PREDIMED-UNAV with *n* = 116 (27), and RESMENA with *n* = 44 (28), whose providers are gratefully acknowledged.

The second study involved 269 adult participants from the OBEKIT study with *n* = 203 (29) and the NormoP study with *n* = 66 (30). Study design, characteristics, inclusion and exclusion criteria of each of these studies have been previously described. This research was carried out in accordance with the recommendations of the Research Ethics Committee of the University of Navarra (CEI-UN, Pamplona, Spain). All subjects gave written informed consent in accordance with the Declaration of Helsinki. The protocol was approved by the Research Ethics Committee of the University of Navarra (CEI-UN, Pamplona, Spain), except for GEDYMET, which was approved by the ethics committee of the School of Medicine, Pontificia Universidad Católica de Chile (Santiago, Chile).

### Study Variables

Anthropometric (waist circumference), blood pressure and biochemical measurements [glucose, HOMA-IR, HDL-cholesterol, triglycerides, TyG index, C-reactive protein (CRP)] were retrieved from databases of the aforementioned studies following validated protocols. The HOMA-IR index was calculated as fasting insulin (μUI/mL) × fasting glucose (mg/dL)/405. The triglycerides-glucose (TyG) index was calculated as Ln(triglycerides [mg/dL] x glucose [mg/dL]/2) as an indicator of IR (31). Body composition in the second study was assessed by Dual energy X-ray absorptiometry (DXA) according to the manufacturer′s instructions (Lunar iDXA, enCORE 14.5, GE Healthcare, Madison, WI, USA). Visceral adipose tissue mass was assessed with the CoreScan application for the software enCORE (GE Healthcare, Madison, WI, USA).

### DNA Extraction and DNA Methylation Analysis

Venous blood samples were collected in EDTA tubes. Genomic DNA was extracted from peripheral white blood cells using the MasterPure™ DNA Purification kit (Epicenter, Madison, WI) and quantified with Pico Green dsDNA Quantitation Reagent (Invitrogen, Carlsbad, CA). In order to convert cytosine into uracil, high-quality DNA samples (500 ng) were treated with bisulfite using the EZ-96 DNA Methylation Kit (Zymo Research Corporation, Irvine, CA) according to the manufacturer's protocol. Infinium Human Methylation 450K BeadChip technology (Illumina, San Diego, CA, USA) was employed to measure DNA methylation levels in all the studies, except for OBEKIT and NormoP studies, which were performed with Infinium MethylationEPIC BeadChip (Illumina). The analyses were conducted in the Unidad de Genotipado y Diagnóstico Genético from Fundación Investigación Clínico de Valencia, as detailed elsewhere (32).

### Treatment of Methylation Raw Data

Beta-values have been used to assess methylation levels in order to estimate the methylation degree using the ratio of the methylation probe intensity and the overall intensity, corresponding to the percentage of methylation on a specific site (33). Intensity data were obtained using the ChAMP package for R v.1.11.0 (34) as described elsewhere (35). Normalization of methylation samples from both studies was performed with the function preprocessNoob of Minfi package from Bioconductor. This function allows background correction with dye-bias normalization (36).

### Estimation of Epigenetic Age Acceleration

Epigenetic age accelerations were defined as the residual (difference between the observed and the predicted value) from regressing the epigenetic age on chronological age (AgeAcc for DNAmAge method and AgeAccGrim for GrimAge method) as described by (37). After pre-processing, epigenetic age accelerations were calculated using two different methods available on the website DNA methylation Age Calculator (https://dnamage.genetics.ucla.edu/home) (16). The first method, denominated DNAmAge and developed by Horvath (16), was designed to calculate the epigenetic age (DNAm age) from human samples profiled with the Illumina Infinium 450K platform. This procedure was based on the DNA methylation levels of 353 CpGs. The second method, denominated GrimAge, was recently created by Horvath and collaborators (18). Briefly, this epigenetic clock was a method of epigenetic age prediction based on a linear combination of chronological age, sex, and DNAm-based surrogate biomarkers for seven plasma proteins and smoking pack-years. As indicated by Horvath on the DNA methylation Age Calculator website, samples with a correlation coefficient <0.8 with the gold standard (defined by averaging the beta values across the samples from the largest blood data set) were excluded (data provided by the website application).

### Statistical Analysis

Variables with skewness >1 were log2-transformed [glucose, HOMA-IR, triglycerides and CRP (adding +1 to avoid the logarithm of zero)] as previously described (38). Age accelerations (AgeAcc and AgeAccGrim) from the first study were correlated (Pearson's r) with different variables including waist circumference, glucose (log2), HOMA-IR (log2), HDL-cholesterol, triglycerides (log2), systolic and diastolic blood pressure and the TyG index. Differences between women and men in the second study (OBEKIT + NormoP) were calculated using two-independent samples Student *t*-test. AgeAccGrim data from the second study were correlated (Pearson's r) with different anthropometric measurements determined by DXA (fat mass, lean mass, trunk fat mass, android fat mass, gynoid fat mass, visceral adipose tissue mass), the metabolic syndrome variables as measured in the MENA study, and the CRP (log2) as a marker of inflammation. Multiple linear regression models between AgeAccGrim and some variables from the second study (model 1: visceral adipose tissue mass and TyG index, model 2: HDL-cholesterol and CRP) were fitted, separating by sex. Mediation by TyG index in the relationship between AgeAccGrim and visceral adipose tissue mass DXA in men was assessed using structural equation modeling following the Zhao et al. approach (39).

*p*-values were considered statistically significant if p <0.05. Statistical calculations were performed with Stata version 12.1 (StataCorp 2011, College Station, TX, USA).

## Results

### Correlations in the First Study Sample

Anthropometric, biochemical and blood pressure measurements of the first study are reported (Table 1).

**Table 1:** First study phenotypical and clinical characteristics.

	**First study**	
**Variable**	***n***	Values^*^
Sex (females)	366	234 (63.9%)
Age (years)	366	47.3 (15.4)
Waist circumference (cm)	365	94.2 (16.5)
Glucose (mg/dL)	335	105 (33)
HOMA-IR	224	2.7 (2.5)
HDL-cholesterol (mg/dL)	339	54 (14)
Triglycerides (mg/dL)	339	125 (77)
TyG index	334	8.6 (0.7)
Systolic blood pressure (mmHg)	336	132 (22)
Diastolic blood pressure (mmHg)	336	78 (12)

* *Values are represented as Mean (SD) except for sex which is represented as number of females(%)*.

Correlations between age accelerations measured by Horvath (AgeAcc) or by Lu et al. (AgeAccGrim) and the anthropometric, biochemical and blood pressure variables were performed for participants from the first study (Table 2). Results showed statistically significant associations between both age accelerations and all the anthropometric and biochemical measurements, except for glucose and triglycerides, which were only significant for AgeAccGrim. In addition, AgeAccGrim showed higher correlation coefficients and statistical significance with most of the variables, while no statistical associations were found with blood pressure.

**Table 2:** Correlations in the first study sample between epigenetic age accelerations calculated by two different methods and anthropometric, biochemical, and blood pressure variables.

		**AgeAcc**		**AgeAccGrim**	
**Variable**	***n***	***r***	***p***	***r***	***p***
Waist circumference (cm)	365	0.22	** <0.001**	0.37	** <0.001**
log2(Glucose) (mg/dL)	335	0.09	0.104	0.19	** <0.001**
log2(HOMA-IR)	224	0.15	**0.029**	0.41	** <0.001**
HDL-cholesterol (mg/dL)	339	−0.19	** <0.001**	−0.31	** <0.001**
log2(Triglycerides)(mg/dL)	339	0.11	0.051	0.23	** <0.001**
TyG index	334	0.11	**0.042**	0.26	** <0.001**
Systolic blood pressure (mmHg)	336	0.10	0.062	0.09	0.118
Diastolic blood pressure (mmHg)	336	0.08	0.125	−0.04	0.493

*A significant p-value is considered p < 0.05 (in bold)*.

### Correlations in the Second Study Sample

Anthropometric, biochemical, and blood pressure measurements of the second study are reported (Table 3). Since several variables showed differences between women and men (Table 3), further analyses were performed separating by sex.

**Table 3:** Second study phenotypical and clinical characteristics.

	**Second study**						
**Variable**	**Total**		**Women**		**Men**		**Women vs. Men**
	***n***	**Values^**a**^**	***n***	**Values^**a**^**	***n***	**Values^**a**^**	**p^**b**^**
Sex (females)	268	189 (70.5)					
Age (years)	268	44.8 (10.2)	189	44.7 (10.4)	79	44.8 (9.7)	0.946
Waist circumference (cm)	268	95.6 (15.2)	189	92.7 (14.7)	79	102.7 (14.0)	** <0.001**
Fat mass DXA (kg)	267	27.7 (17.1)	189	28.0 (17.6)	78	16.3 (9.9)	0.693
Lean mass DXA (kg)	267	36.2 (22.4)	189	31.9 (19.1)	78	27.0 (16.2)	** <0.001**
Trunk fat mass DXA (kg)	267	15.0 (15.2)	189	14.4 (9.3)	78	46.6 (26.2)	0.143
Android fat mass DXA (kg)	267	2.7 (1.6)	189	2.6 (1.5)	78	3.0 (1.8)	**0.041**
Gynoid fat mass DXA (kg)	267	4.6 (2.9)	189	4.9 (3.1)	78	3.9 (2.4)	**0.012**
Visceral adipose tissue mass DXA (kg)	264	1.1 (0.9)	186	0.9 (0.6)	78	1.8 (1.1)	** <0.001**
Glucose (mg/dL)	268	93 (11)	189	91 (10)	79	97 (11)	** <0.001**^**c**^
HOMA-IR	268	1.7 (1.2)	189	1.6 (1.2)	79	1.8 (1.4)	0.513^c^
HDL-cholesterol (mg/dL)	268	57 (13)	189	60 (13)	79	49 (10)	** <0.001**
Triglycerides (mg/dL)	268	93 (50)	189	85 (39)	79	113 (65)	** <0.001**^**c**^
TyG index	268	8.3 (0.5)	189	8.2 (0.5)	79	8.5 (0.5)	** <0.001**
C-reactive protein (μg/mL)	268	2.5 (3.6)	189	2.7 (3.9)	79	2.0 (2.7)	0.178^c^
Systolic blood pressure (mmHg)	260	124 (17)	184	120 (15)	76	133 (16)	** <0.001**
Diastolic blood pressure (mmHg)	260	77 (11)	184	75 (10)	76	81 (11)	** <0.001**

a Values are represented as Mean (SD) except for sex which is represented as number of females(%).

b Values obtained by Student t-test comparing women and men. A significant p-value is considered p < 0.05 (in bold).

c *The calculations have been performed with the log-transformed variable in base 2. For C-reactive protein, +1 was added to avoid forming the logarithm of zero*.

Correlations in the second study for both sexes and separated by sex were calculated with AgeAccGrim (Table 4) since it was the epigenetic age acceleration that showed better correlation coefficients and significance with many variables analyzed in the first study (Table 2). Correlations in the second study were statistically significant for the same variables as those obtained from the first study, except for HOMA-IR, which was not significant in the OBEKIT+NormoP study. Moreover, both CRP and many of the anthropometrical measurements assessed by DXA were also significantly associated with AgeAccGrim. When analyzing by sex, women showed statistically significant correlations between AgeAccGrim and HDL-cholesterol or CRP, whereas in men the identified associations were significant with visceral adipose tissue mass measured by DXA, triglycerides and TyG index. In order to analyse whether these significant correlations were different between both sexes, comparison of both correlation coefficients was performed. Significant differences were only found for triglycerides and TyG index (Table S1).

**Table 4:** Correlations in the second study between epigenetic age acceleration estimated by AgeAccGrim and anthropometric, body composition, biochemical and blood pressure variables.

	**AgeAccGrim**								
	**Total**			**Women**			**Men**		
**Variable**	***n***	***r***	***p***	***n***	***R***	***p***	***n***	***r***	***p***
Waist circumference (cm)	268	0.17	**0.006**	189	0.09	0.208	79	0.15	0.200
Fat mass DXA (kg)	267	0.07	0.256	189	0.07	0.343	78	0.10	0.385
Lean mass DXA (kg)	267	0.20	**0.001**	189	0.06	0.407	78	0.10	0.398
Trunk fat mass DXA (kg)	267	0.13	**0.029**	189	0.09	0.221	78	0.12	0.312
Android fat mass DXA (kg)	267	0.13	**0.032**	189	0.08	0.262	78	0.13	0.250
Gynoid fat mass DXA (kg)	267	0.004	0.942	189	0.06	0.435	78	0.09	0.448
Visceral adipose tissue mass DXA (kg)	264	0.21	** <0.001**	186	0.13	0.073	78	0.23	**0.043**
log2(Glucose) (mg/dL)	268	0.17	**0.006**	189	0.11	0.146	79	0.13	0.243
log2(HOMA-IR)	268	0.09	0.165	189	0.08	0.283	79	0.05	0.672
HDL-cholesterol (mg/dL)	268	−0.26	** <0.001**	189	−0.21	**0.004**	79	−0.16	0.161
log2(Triglycerides)(mg/dL)	268	0.19	**0.002**	189	0.07	0.356	79	0.35	**0.001**
TyG index	268	0.22	** <0.001**	189	0.08	0.245	79	0.34	**0.002**
log2(C-reactive protein+1) (μg/mL)	268	0.14	**0.020**	189	0.17	**0.017**	79	0.21	0.068
Systolic blood pressure (mmHg)	260	0.07	0.249	184	−0.03	0.645	76	0.09	0.453
Diastolic blood pressure (mmHg)	260	0.06	0.350	184	0.01	0.885	76	−0.03	0.789

*A significant p-value is considered p< 0.05 (in bold)*.

### Linear Regression Models in the Second Study

Linear regression models were then fitted for the second study separated by sex (Table 5). Two different models were applied: model 1 included visceral adipose tissue mass measured by DXA and TyG index, whereas model 2 included HDL-cholesterol and CRP. Model 1 was statistically significant for men, but not for women; on the contrary, model 2 was statistically significant for women, but not for men.

**Table 5:** Linear regression models of age acceleration AgeAccGrim separated by sex.

	**AgeAccGrim**					
	**Women**			**Men**		
	**β**	***p***	$R_{\text{adj}}^{2}$	**β**	***p***	$R_{\text{adj}}^{2}$
**Model 1**		0.199	0.01		**0.007**	0.10
Visceral adipose tissue mass DXA (kg)	0.803	0.158		0.037	0.944	
TyG index	0.114	0.882		2.534	**0.017**	
**Model 2**		**0.004**	0.06		0.050	0.03
HDL-cholesterol (mg/dL)	−0.054	**0.022**		−0.039	0.436	
log2(C-reactive protein+1) (μg/mL)	0.474	0.092		0.740	0.163	

*A significant p-value is considered p < 0.05 (in bold)*.

### Mediation Model in the Second Study

**Figure 1:**
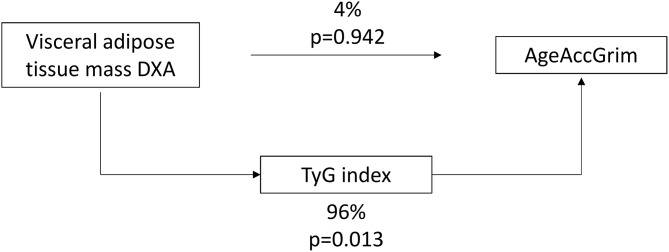
TyG index-mediated relationship between visceral adipose tissue mass measured by DXA and age acceleration GrimAge in men.

Since visceral adiposity has been suggested as the key point for the onset of IR, a possible mediation by TyG index in the relationship between visceral adipose tissue mass DXA and AgeAccGrim was assessed in men. Results showed that TyG index was mediating the majority of the effects of visceral adipose tissue mass accumulation on age acceleration (Figure 1).

## Discussion

Aging is an irreversible and progressive process that occurs from birth (40). Many theories have been proposed about aging (41), including wear-and-tear theories, the free radical hypothesis of aging, the cross-linking theory, and the neuroendocrine theory, among others (40). Interestingly, newer studies have suggested a role of epigenetic processes in lifespan regulation that should be further investigated (42). In this context, aging-related DNA methylation has been hypothesized as a biomarker that predicts both cellular age and chronological age (42).

Fat accumulation, and specifically, excessive visceral fat deposition, is common in elderly individuals. Excessive visceral fat deposition usually increases with age, as it is related to obesity, diabetes, cardiovascular diseases, cancer and fatty liver disease (11, 43). Visceral fat has an important role in the onset of IR (44). Subjects with accumulation of abdominal fat presented enhanced lipolysis and alteration of the flux of free fatty acids (44). The rise of circulating free fatty acids in turn increases hepatic gluconeogenesis and glycogenolysis, as well as increasing insulin secretion by the pancreas, resulting in hyperglycaemia (45). In addition, in the liver, there is a higher production of triglycerides and less insulin breakdown, leading to hyperinsulinemia (45). Hyperglycaemia and hyperinsulinemia promote IR (46). Interestingly, the TyG index has been determined as a useful marker of IR (44). Indeed, it has been demonstrated as a better marker for the diagnosis of metabolic syndrome than HOMA-IR, since it involves measures of both triglycerides and glucose (47).

Aging is an important contributor to metabolism and health impairments, increasing the risk for several diseases (48). Numerous mechanisms related to longevity and age-related metabolic dysfunctions occur in adipose tissue (48). Therefore, the investigation of the relationships between adipose tissue, IR and aging and the role of epigenetics in this process is a challenging task that could provide new understanding about the putative interactions among them.

The assessment of aging has received attention because chronological age is sometimes different from biological and metabolic age (49). Therefore, different methods based on epigenetic measurements have been designed for that purpose: an estimator developed by Hannum et al. (50) based on 71 CpGs in leukocytes; DNAmAge clock created by Horvath (16) based on 353 CpGs in different tissues; PhenoAge designed by Levine et al. (17) for predicting lifespan based on regressing a phenotypic measure of mortality risk on CpGs; and the newest one, GrimAge, developed by Lu et al. (18) using DNAm-based biomarkers for seven plasma proteins and smoking. GrimAge has been reported as a better predictor of lifespan than currently available DNAm-based predictors (18). Studies with these epigenetic clocks have demonstrated epigenetic age acceleration in obesity (37, 51), diabetes (51, 52), non-alcoholic fatty liver disease (53) and different metabolic and inflammatory biomarkers (54). In this sense, epigenetic age acceleration could be defined as the aging of epigenetic DNA regulation, which may lead to longevity functions impairments earlier than expected.

A number of factors, such as sex, could mediate the interactions between epigenetic aging and adiposity biomarkers, since it is known that visceral fat accumulation is more common in men than in women (10). Furthermore, sex differences elicited by sexual hormones affect this adiposity dimorphism. In this context, estrogens may direct the expansion of fat through the increase of adiposity progenitor cells (hyperplasia) as well as regulate the vascular supply into adipose tissues (55) with impact on epigenetic mechanisms (56).

Indeed, our investigation demonstrated that sex is important in the analysis of the relationship between epigenetic age acceleration and adiposity biomarkers. Men showed a tighter association of epigenetic age acceleration with visceral adipose tissue mass and the TyG index, whereas HDL-cholesterol and CRP were associated with epigenetic age acceleration in women. However, we cannot conclude that the correlations observed were statistically different between sex groups, except for TyG index. These results are also in agreement with the fact that aging is usually accompanied by a deterioration of metabolic markers (57, 58). Furthermore, we found that the relationship between visceral adipose tissue and epigenetic age acceleration in men is mainly mediated by the TyG index. Thus, the accumulation of abdominal fat, through TyG index mediation, suggest an older biological age in men than the expected. This result is in accordance with the fact that visceral adipose tissue contributes to the development of IR (44) and that both are influencing aging processes (37, 59).

Other possible factors that might influence epigenetic aging are HDL-cholesterol or CRP, as described in our research. HDL-cholesterol relationship with epigenetic age acceleration was negative, thereby suggesting a deceleration of biological age with higher levels of this lipoprotein. Thus, HDL-cholesterol might modulate epigenetic aging processes due to its antiatherogenic effects such as the removal of lipid deposits, which is accompanied by the reduction of cytotoxic effects (60). Furthermore, HDL reduces the oxidative stress in plasma and cellular compartments, and the signaling pathways which it participates in are interconnected with stress response and survival pathways (60). On the other hand, higher CRP levels were related to accelerated biological age, suggesting a negative influence of inflammation on aging-related epigenetic marks. Indeed, CRP is a molecule involved in inflammatory and immune processes (61), which has been associated with aging-related diseases such as diabetes, cardiovascular diseases and hypertension (62). Additionally, previous research showed that after adjusting for age, sex, body mass index, lipid levels and smoking status, increased levels of CRP were associated with cognitive and poor physical performances and reduced survival (63).

This research did have some limitations. The ideal tissue assessed in this study would have been adipose tissue since the relationship was established between visceral adipose tissue mass and age acceleration. However, several studies have demonstrated that peripheral blood is a valid non-invasive alternative tissue that reflects metabolic and inflammatory pathways and serves as a surrogate for assessing methylation (64–67). Furthermore, DNAm age performs well in blood, (16) and GrimAge has been developed with methylation data from blood samples (18). Another possible limitation is that methylation samples in the MENA and in the OBEKIT+NormoP studies were assessed with different Illumina arrays (Infinium 450K BeadChip and EPIC, respectively). Nevertheless, McEwen *et al*. revealed that both methods were equivalent in the determination of epigenetic age (68). Moreover, it must be noted that the percentage of contribution of our variables to epigenetic age acceleration is at maximum of 10%. Thus, there must be other factors influencing the progression of aging.

In summary, our research has revealed that the mechanisms that affect epigenetic age acceleration are different in women and men. Whereas, in men the accumulation of visceral adipose tissue accelerates the epigenetic age through mechanisms mostly mediated by IR, mechanisms in women seemed to be more related to HDL-cholesterol and CRP. These findings support the hypothesis that obesity and metabolic syndrome features, intimately related to adiposity and dyslipidaemia, are associated with accelerated aging effects (43), although in a different manner for each sex. Further research about the relationship between epigenetic age acceleration and disease markers will allow for a better understanding of the molecular mechanisms involved in the development of age-related diseases. This knowledge is needed to create new useful clinical and public health tools based on DNA methylation biomarkers and epigenetic age acceleration. Furthermore, sex differences concerning the factors influencing epigenetic age acceleration should be taken into account in future development of precision strategies and management of healthy aging.

## Data Availability

The data of the first study have been deposited in NCBI's Gene Expression Omnibus (69) and are accessible through GEO Series accession number GSE115278 (https://www.ncbi.nlm.nih.gov/geo/query/acc.cgi?acc=GSE115278). The remaining data will be made available by the authors, without undue reservation, to any qualified researcher.

## Ethics Statement

This study was carried out in accordance with the recommendations of the Research Ethics Committee of the University of Navarra (CEI-UN, Pamplona, Spain). All subjects gave written informed consent in accordance with the Declaration of Helsinki. The protocol was approved by the Research Ethics Committee of the University of Navarra (CEI-UN, Pamplona, Spain), except for GEDYMET, which was approved by the Ethics committee of the School of Medicine, Pontificia Universidad Católica de Chile (Santiago, Chile).

## Author Contributions

AA performed data analysis and wrote the first version of the paper. JS helped with data interpretation. MG-G helped with the statistical analysis. J-IR-B, FM, and JM supervised data analysis and helped with interpretation and with manuscript elaboration, as well as provided the conceptual design and financial support. All authors read and approved the final manuscript.

### Conflict of Interest Statement

The authors declare that the research was conducted in the absence of any commercial or financial relationships that could be construed as a potential conflict of interest.
