# Supplementary material for: Interaction Among Sex, Aging, and Epigenetic Processes Concerning Visceral Fat, Insulin Resistance, and Dyslipidaemia
Source: Front Endocrinol (Lausanne). 2019 Jul 17;10:496. doi: 10.3389/fendo.2019.00496 (PMC6653993; doi:10.3389/fendo.2019.00496)
Supplement: Supplementary file 1 [file Table_1.DOCX]

Supplementary Material

# Supplementary Tables

**Table S1:** Differences in the correlations (AgeAccGrim vs. Variable) between sex groups in the second study

| **Variable** | **Women** | | **Men** | | **p-value*** |
| --- | --- | --- | --- | --- | --- |
|  | **n** | **r** | **n** | **r** |  |
| Visceral adipose tissue mass DXA (kg) | 186 | 0.13 | 78 | 0.23 | 0.451 |
| HDL-cholesterol (mg/dL) | 189 | -0.21 | 79 | -0.16 | 0.704 |
| log2(Triglycerides) (mg/dL) | 189 | 0.07 | 79 | 0.35 | 0.030 |
| TyG index | 189 | 0.08 | 79 | 0.34 | 0.044 |
| log2(C-reactive protein+1) | 189 | 0.17 | 79 | 0.21 | 0.760 |

*p-value is calculated using a Z test on the Fisher-transformed coefficients.
